# A transcriptomic survey of the impact of environmental stress on response to dengue virus in the mosquito, *Aedes aegypti*

**DOI:** 10.1371/journal.pntd.0006568

**Published:** 2018-06-11

**Authors:** David S. Kang, Martin S. Barron, Diane D. Lovin, Joanne M. Cunningham, Matthew W. Eng, Dave D. Chadee, Jun Li, David W. Severson

**Affiliations:** 1 Department of Biological Sciences, University of Notre Dame, Notre Dame, IN, United States of America; 2 Department of Applied and Computational Mathematics and Statistics, University of Notre Dame, Notre Dame, IN, United States of America; 3 Department of Life Sciences, University of the West Indies, Saint Augustine, Trinidad and Tobago; University of Glasgow, UNITED KINGDOM

## Abstract

Populations of *Aedes aegypti* naturally exhibit variable susceptibility to dengue viruses. This natural variation can be impacted by nutritional stress resulting from larval-stage crowding, indicating the influence of environment components on the adult mosquito immune response. In particular, larval crowding was previously shown to reduce the susceptibility of adult females of a Trinidad field isolate of *A*. *aegypti* to the dengue serotype 2 (JAM1409) virus. Here, we present the first whole transcriptome study to address the impact of environmental stress on *A*. *aegypti* response to dengue virus. We examined expression profiles of adult females resulting from crowded and optimum reared larvae from the same Trinidad isolate at two critical early time points—3 and 18 hours post dengue virus infected blood meal. We exposed specimens to either a dengue or naïve blood meal, and then characterized the response in ten gene co-expression modules based on their transcriptional associations with environmental stress and time. We further analyzed the top 30 hub or master regulatory genes in each of the modules, and validated our results via qRT-PCR. These hub genes reveal which functions are critical to the mechanisms that confer dengue virus refractoriness or susceptibility to stress conditioned *A*. *aegypti*, as well as the time points at which they are most important.

## Author summary

Dengue is a neglected tropical disease which infects about 390 million people a year, particularly in areas of poverty. Currently, control of the primary dengue virus vector mosquito, *Aedes aegypti*, remains the only effective method of preventing the proliferation and spread of dengue virus. Contemporary control programs largely fail to consider the effects of environmental conditions on the ecology and life history traits, including innate immunity of mosquito populations. Here, we provide global, transcriptional analyses of how crowding-induced environmental stress results in reduced susceptibility of *A*. *aegypti* to dengue virus. This information has potential to inform the accurate conception and implementation of successful control programs. Additionally, the underlying genetic mechanisms responsible for these differences in susceptibility may have application toward either transgenic mosquito population replacement or transmission blocking vaccination approaches to disease prevention.

## Introduction

The *Aedes aegypti* mosquito is the primary global vector of the dengue, chikungunya, Zika, and yellow fever viruses. Dengue virus in particular is known to infect about 390 million people a year, with approximately 96 million individuals a year afflicted with clinical symptoms [1]. Presently, control of mosquito populations is the only strategy available for the suppression of many of these arboviruses [1, 2]. Unfortunately, the rise of insecticide resistant populations of mosquitoes has proven to impede traditional vector control strategies [3]. As such, investigation into the genetic and environmental basis underlying differences of resistance of mosquito populations to infection may inform efforts to develop novel future disease transmission disruption methods. Here, we provide the first comprehensive transcriptional survey of the impact of environmental stress on *A*. *aegypti* response to dengue virus (DENV) infection.

In the wild, *A*. *aegypti* are opportunistic ovipositors and are known to utilize water-filled vessels rather indiscriminately, often resulting in high levels of larval stress due to prevailing environmental conditions and competition for nutritional resources. These stressors have been demonstrated to impact a suite of life history traits including adult body size, reproductive fitness, longevity, blood-feeding, the ability of the innate immune system to resist viruses such as DENV and vector competence [4–9]. Interestingly, this adaptive plasticity has been found to diminish in laboratory populations of *A*. *aegypti* over time, with a key study demonstrating the loss of variability in heritability with regard to body size declining over generations of captivity [10]. This study concluded that body size plasticity is conserved in the field due to the prevalence of variable environmental stress during larval development and is conserved by balancing selection. We hypothesized that other life history traits, including the innate immune response, would be likewise impacted by environmental stress.

Previous studies have already established that innate differences of susceptibility between *A*. *aegypti* strains to DENV are reflected in transcriptional activity [11]. In particular, differential expression of classic immune pathways such as Toll [12] and JAK/STAT [13] pathways have been implicated in comparative and functional investigations of response to virus infection. Despite this, there are a wealth of other genes, including those encoding vacuolar ATPases [14] and serine proteases [15, 16], that have been shown to be highly differentially expressed in response to DENV infection, but their exact role in determining eventual vector competence are still poorly understood.

Nevertheless, these previous studies have established a foundation from which we may now consider the influence of environmental stressors on mosquito innate immunity and how this may translate to field populations. Here, we utilized larval rearing conditions to simulate environmental stress and then characterized which functions were conserved as general responses to DENV and which are modulated by stress, revealing those pathways from our modules that are most promising for practical application in disease control. We previously demonstrated that rearing mosquitoes of shared genetic background under optimal laboratory conditions vs. simulated field conditions via density induced nutritional stress impacts their susceptibility to DENV, with stressed mosquitoes exhibiting significantly lower dissemination rates (18.1%) than optimally reared individuals (37.5%) [17]. In that study, females testing negative for disseminated head infection also failed to exhibit significantly increased midgut infection, indicating the mechanism of action is likely an early preventative midgut infection barrier rather than a post-infection midgut escape barrier. These results were supported by literature demonstrating that exposure to DENV and subsequent endocytotic ingress of the virus occurs quickly (<30 min) [18, 19], yet we know very little about the factors that link environmental stress to the refractory phenotype.

Here, we reasoned that environmental stress further impacts the immune response in *A*. *aegypti*, resulting in altered susceptibility to DENV infection. In this study we tested whether or not increased resistance of stressed mosquitoes to DENV is mediated by differential transcription at 3 and 18 hours post DENV exposure. We compared and evaluated expression in stressed and optimally reared adult females after both DENV infected and uninfected blood feeding. We identified extensive modular transcriptional networks underlying the above mentioned environmentally driven variability, as well as the master regulatory or hub genes that represent key transcriptional regulators responsible for the appropriate innate immune response.

## Materials and methods

### Mosquito rearing and maintenance

Experiments were performed on F_3_ progeny from a colony of *Aedes aegypti* established at the University of Notre Dame using ovitrap collected eggs from Curepe, Trinidad [17]. Rearing chambers were kept at 26°C, 85% relative humidity with a 16 hour light:8 hour dark (L:D) cycle with a 30 minute crepuscular period as per standard protocol [17]. Larvae were then raised under density induced nutritionally stressed or optimal conditions [6, 17]. 75 first instar larvae were placed in 1 liter of distilled water for the optimal treatment, and 750 larvae in the same volume of water, for the stressed treatment. One day after hatching, larvae were provided 75 mg of bovine liver powder (MP Biomedicals, LLC), then on day two 0 mg, 75 mg on day three, 113 mg on day four and finally 150 mg on day five. Upon pupation, individuals were transferred to 500 mL of fresh water in 20x20x30 cm mesh cages until adult eclosion. Mosquitoes were then maintained on 10% sucrose saturated cotton balls until 7 days post eclosion. As previously described [17, 20–22], in order to assess the efficacy of the treatments, the right wings of thirty adult females from each treatment were then assessed for wing length, as a proxy for body size. The remaining mosquitoes were then transferred to 500 mL paper cups and starved for 24 hours prior to infectious blood feeding. Rat blood feeding was utilized for mosquito colony maintenance. Animal use was as described in the Guide for the Care and Use of Laboratory Animals by the National Institutes of Health using a protocol approved by the University of Notre Dame Institutional Animal Care and Use Committee (Study #11–036). Females were then briefly anesthetized with CO_2_ for sorting and then maintained as described above until RNA extractions were performed.

### Cell culture and dengue virus infection

Cell culture and mosquito infections were performed as previously described [22]. Briefly, *Aedes albopictus* C6/36 cells were maintained on 10% fetal bovine serum (FBS) at 28°C to near confluence (~80%) in 75 cm^3^ flasks before inoculation with DENV2 (strain JAM1409) at a multiplicity of infection (MOI) of 0.1. Infected cells were then incubated for 7 days on 2% FBS infused L-15 media at 28°C, before collection of the supernatant by centrifugation at 2,500 RPM for 10 minutes, then freezing at -80°C. For the infectious blood meals, frozen DENV2 supernatant (TCID_50_ of 10^6.5)^ was thawed and mixed with defibrinated sheep blood (Colorado Serum Company) in an equal ratio before being offered to female mosquitoes using an artificial glass membrane feeder with a rat skin membrane at 37°C. Stressed and optimal exposure to dengue were performed simultaneously from the same aliquots of DENV2. Infection for RNAseq analysis was performed concurrently with the previously reported dissemination experiment [17], utilizing cohort specimens and the same aliquot of DENV2 for consistency. Negative control individuals followed the same protocol with an uninfected C6/36 cell culture.

### Library preparation and sequencing

RNA samples were collected from whole bodies of blood fed females at 3 hours and 18 hours post DENV exposure via the RNAeasy Kit (Qiagen) as per manufacturer instructions. A total of 5 samples of 5 pooled females were extracted per each of the 8 treatments. Blood meals were first removed with micro syringe needles with care to leave the midgut intact. RNA was quantified by NanoDrop spectrophotometer (Thermo Fisher Scientific), then verified via Qubit fluorometric quantitation (Thermo Fisher Scientific), and Kapa Library Quantification qPCR assays (Illumina) with RNA integrity assessed via Bioanalyzer DNA 7500 chip (Agilent). The three highest quality samples from each treatment were then selected for Truseq RNA Library Preparation (Illumina) by the Genomics and Bioinformatics Core Facility (http://genomics.nd.edu/genomics-bioinformatics-core-facility) at the University of Notre Dame before transcriptomic analyses on a NextSeq 500 (Illumina).

### Preliminary data analysis and preparation

Run statistics were assessed with FASTQC before read alignment and differential expression analysis was carried out using EdgeR [23]and DESeq2 [24]. Transcripts were compared to the reference genome of *A*. *aegypti* AaegL3.4 (www.vectorbase.org) [25]. Differential expression was calculated by comparing the DENV2 and control samples from each of the experimental conditions (3 hour optimum, 3 hour stressed, 18 hour optimum, 18 hour stressed). Only genes, uniquely or significantly differentially expressed, false discovery rate adjusted p-value (q-value) < 0.001, in the experimentals when compared to the controls were used for the remaining analyses. DESeq2 was then used to normalize the raw count data for the 24 DENV2 samples, after which the data was transformed as log(x+1) to stabilize variances.

### WGCNA analysis

Data was then analyzed via weighted gene correlation network analysis (WGCNA) in order to reduce the high dimensional data into a scale-free network [26] as previously described [14]. A soft-threshold was established with the graph leveling at 10 with an R^2^ value of approximately 0.62%. Modules were then identified using the dynamic cut method from the WGCNA package with the deepSplit parameter set to 1 and a minimum module size of 25. Dissimilarity between eigengenes was then calculated (1 minus correlation), before hierarchical clustering via average linkage. Modules which clustered at a merge height of 0.2 or less, corresponding to a correlation of 0.8 or higher, were merged together. Relationships between modules were then visualized in a cluster tree, a multidimensional scaling plot (MDS), module eigengene expression bars and by global-cross talk mapping. The correlation between modules and the experimental treatment and time point was calculated and visualized via heat map.

In order to characterize the properties of each module we performed DAVID (Database for Annotation, Visualization and Integrated Discovery) analysis [27], where genes were classified by biological process, cellular component and molecular function via the GO (Gene Ontology) database. The specific gene function of the top 30 hub genes, those genes representing master regulators with the greatest connectivity to other genes, from each module were further evaluated via the BioMart tool on the Vectorbase website (www.vectorbase.org). Hub genes were then examined for intramodular connection as indicated by individual gene connectivity on the STRING (Search Tool for the Retrieval of Interacting Genes/Proteins) database (www.string-db.org) [28].

### qRT-PCR validation

RNA-seq expression of five genes selected from separate modules (module A, vigalin, AAEL018034; module B, cathepsin B, AAEL007590; module C, APG8, AAEL007162; module E, calponin, AAEL008315; module F, wnt10a, AAEL000600) were validated using quantitative real-time PCR (qRT-PCR). Primers were identified utilizing Primer3Plus (http://www.bioinformatics.nl/cgi-bin/primer3plus/primer3plus.cgi), and were designed to span exon-exon junctions (Supplemental 4). Specimens were reared, pooled, infected, assessed and RNA extracted as described above. Quantification was performed utilizing a Power SYBR Green RNA-to-C_T_ 1-Step Kit (Applied Biosystems) on an ABI 7500 Fast System Sequence Detector System (Applied Biosystems). Expression of target genes were normalized with the endogenous housekeeping gene *ribosomal protein S17* (*RPS17*) [29] using the delta-delta C_T_ (ΔΔC_T_) method [30, 31]. Student’s t-tests were utilized to determine differences in ΔC_T_ between treatments at a threshold of P<0.05.

## Results

### General run statistics

The NextSeq 500 runs yielded a total of 800 million reads passing filter with a minimum of 56 million reads per treatment. Over 95% of the reads were assessed at Q30 or higher (above 99.9% base call accuracy).

### Differential expression

A false discovery rate (FDR) adjusted p-value cut-off of 0.001 yielded a total of 2184 genes differentially expressed between DENV2 infected and naïve blood fed samples. Distribution of genes across treatments and time periods is displayed in Table 1.

**Table 1:** Number of *Aedes aegypti* genes showing differential expression between naïve and DENV2 infected mosquitoes.

Condition	Time	No. of genes	Expression
Optimum	3 hr	2	Up-regulated
Optimum	3 hr	1	Down-regulated
Stressed	3 hr	221	Up-regulated
Stressed	3 hr	818	Down-regulated
Optimum	18 hr	522	Up-regulated
Optimum	18 hr	581	Down-regulated
Stressed	18 hr	165	Up-regulated
Stressed	18 hr	15	Down-regulated

### Gene expression analysis

**Fig 1:**
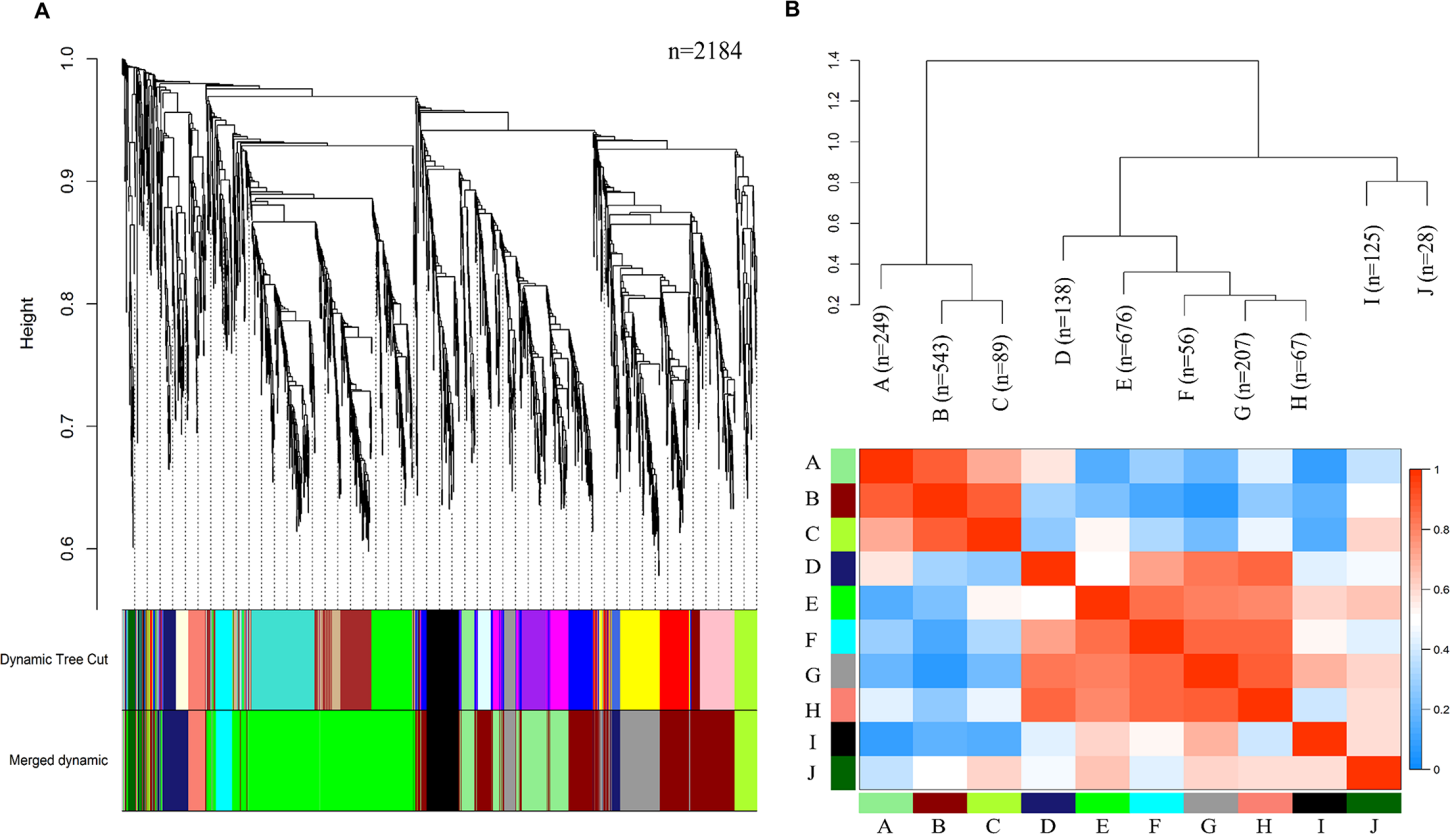
a) Gene dendrogram after dynamic tree cut and after dynamic merge. b) Visualization of the eigengene network representing the relationships among the modules and the trait weight. The top plot shows a hierarchical clustering dendrogram of the eigengenes. The heat map in the bottom plot shows the eigengene adjacency. Merge cut-off was set at 0.2.

**Fig 2:**
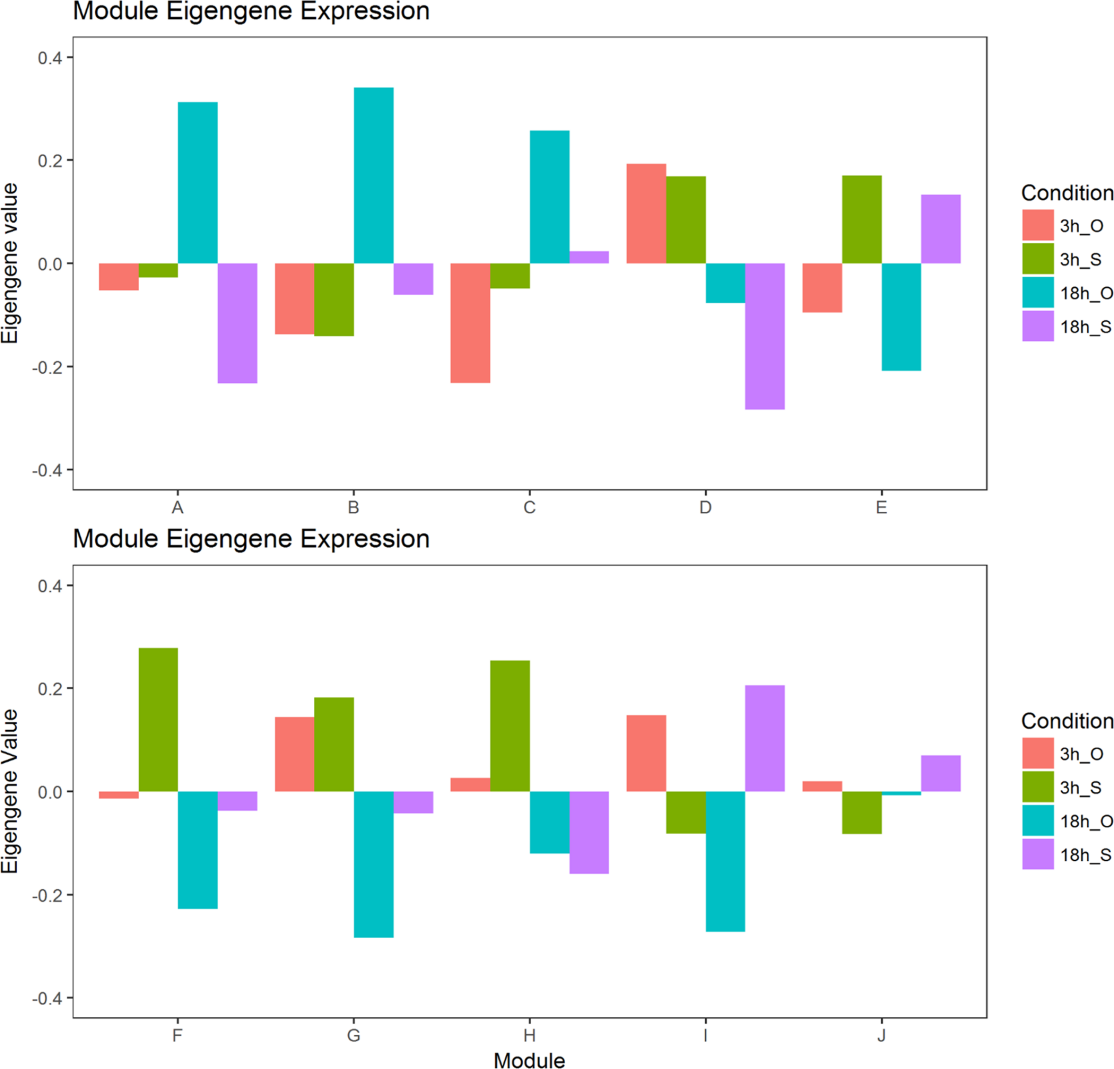
Bar graph representation of the mean eigengene expression patterns for each module for both optimal (O) and stressed (S) treatments at 3 hours (3h) and 18 hours (18h).

WGCNA analyses in R grouped differentially expressed genes with similar expression patterns into modules via hierarchical average linkage clustering. Soft thresholding yielded a graph leveling off around 10 with an R^2^ value of about 0.62%. After noise reduction via transformation of the correlation adjacency matrix into a Topological Overlap Matrix (TOM), conversion to corresponding distance measures and average linkage clustering resulted in 22 modules, containing between 28 to 254 genes, and a null module containing 6 genes remained (Fig 1A). Hierarchical clustering of eigengenes resulted in 10 modules containing 28 to 676 genes, and one null module containing 6 genes (Fig 1B). Each module was comprised of genes with correlated expression patterns. Modules A, B and C share similar expression patterns, and branched distinctly from the group formed by modules D, E, F, G, and H or by the last group containing only modules I and J (Fig 2). The distribution of module genes can be seen in Table 2. Full module gene lists may be found in Supplemental 1.

**Table 2:** Number of genes by corresponding module.

Module	Gene Count
A	249
B	543
C	89
D	138
E	676
F	56
G	207
H	67
I	125
J	28
K	6

**Fig 3:**
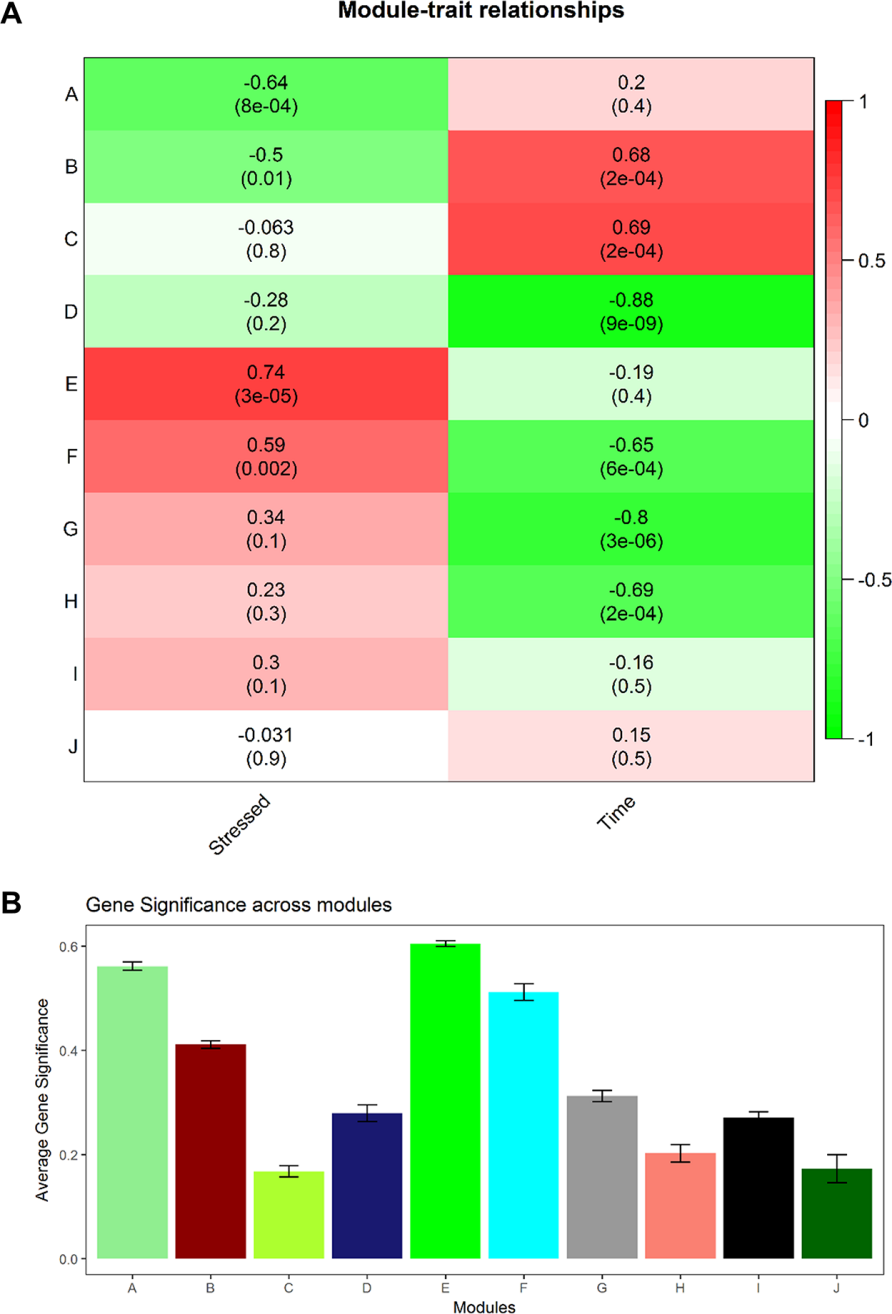
a) Heat map of module-trait relationships. Green correlated with optimal and 3 hour time point, red correlated with stress and 18 hour time point. b) Bar plot of average gene significance across all genes in each module.

Modules were then examined for gene significance and correlation to optimal or stressed treatments as well as relation to other modules (Fig 3), before further analysis on the GO database. Full module DAVID analysis may be found in Supplemental 2. Modules A and B presented strong overall correlation to the DENV susceptible, optimal treatments. Module B was further correlated with the early 18 hour time point, while module A followed suit with a weaker association. Module A revealed enrichment in genes related to DNA replication and initiation, while module B was associated with metabolism, autophagy, apoptosis and fatty acid biosynthesis (Table 3). Module C demonstrated a large shift of optimal eigengene values from 3 hours to 18 hours, and was associated with purine nucleotide biosynthesis, nucleoside metabolism and branched-chain amino acid catabolism. As these modules were all correlated with upregulation in the optimally reared DENV susceptible phenotype, it is likely that the genes contained within are involved with susceptibility to DENV.

**Table 3:** Up to top 4 GO annotation clusters for optimal upregulation modules. P-values adjusted by false discovery rate.

Mod	Clstr	GO Biological Process	p-value	GO Molecular Function	p-value	GO Cellular Component	p-value
A	1	GO:0006270~DNA replication initiation	0.00023	GO:0003678~DNA helicase activity	0.00001	GO:0042555~MCM complex	0.00001
		GO:0005524~ATP binding	0.71542	GO:0003677~DNA binding	0.03613	GO:0005634~nucleus	0.38764
A	2	GO:0006412~translation	0.99986	GO:0003735~structural constituent of ribosome	0.99991	GO:0005840~ribosome	0.99762
A	3	**-**	**-**	GO:0046872~metal ion binding	1.00000	**-**	-
				GO:0003676~nucleic acid binding	1.00000		
A	4	**-**	**-**	**-**	**-**	GO:0016021~integral component of membrane	1.00000
B	1	GO:0006633~fatty acid biosynthetic process	0.07022	GO:0102337~3-oxo-cerotoyl-CoA synthase activity	0.00129	**-**	**-**
				GO:0102336~3-oxo-arachidoyl-CoA synthase activity	0.00129		
				GO:0102338~3-oxo-lignoceronyl-CoA synthase activity	0.00129		
B	2	**-**	**-**	GO:0008234~cysteine-type peptidase activity	0.03762	**-**	**-**
B	3	GO:0008152~metabolic process	0.12891	GO:0003824~catalytic activity	0.11434	**-**	**-**
B	4	GO:0000398~mRNA splicing, via spliceosome	0.37213	**-**	**-**	GO:0005681~spliceosomal complex	0.03948
C	1	-	-	GO:0005524~ATP binding	0.99567068	-	-
C	2	-	-	-	-	GO:0016021~integral component of membrane	0.999934875

**Fig 4:**
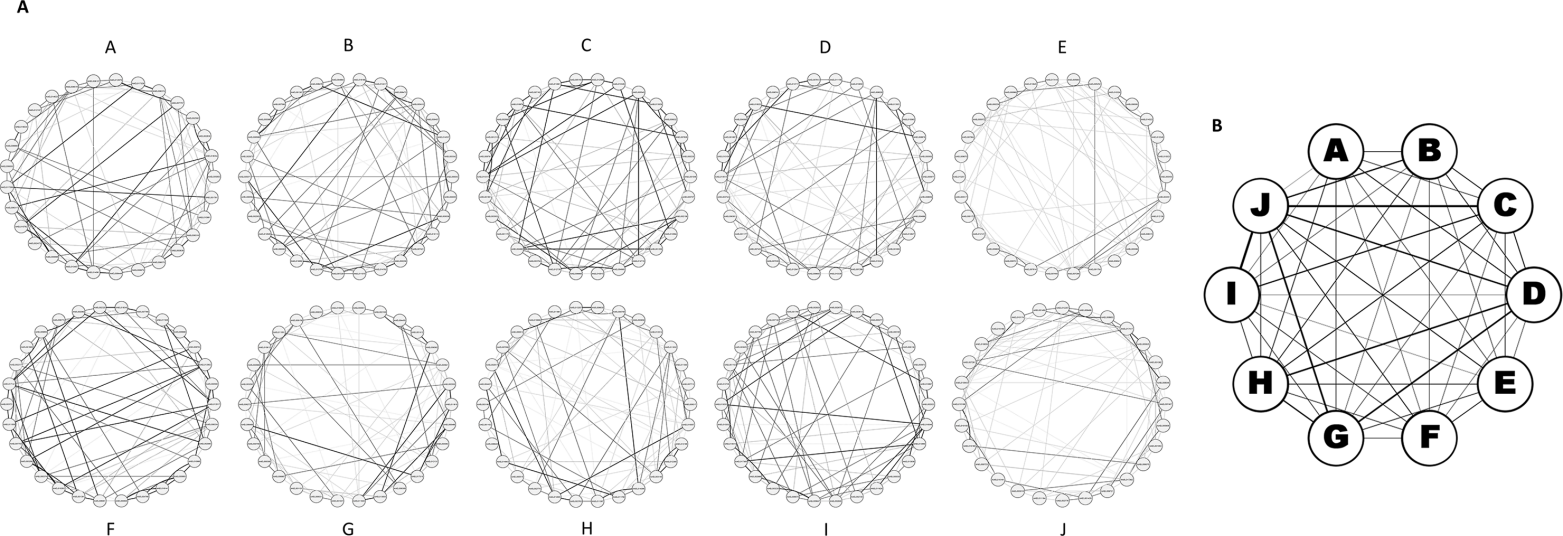
a) Intramodular network of top 30 genes in optimal (A, B, C), stressed (E and F), general response (D, G, H) and time associated modules (I, J). Figure letters correspond with module letter. Thickness of lines represent strength of connections. b) Intermodular network of 10 modules. Thickness of lines represent strength of connections.

Modules E and F presented strong correlation to upregulation in stressed treatments. Module F was correlated with the later 18 hour time point, and module E showed a weaker late time association. Module E was associated with ATP hydrolysis coupled proton transport, oxidative phosphorylation, serine endopeptidase activity, phagosome/lysosome activity as well as enrichment pertaining to transmembrane activity. Similarly, module F exhibited enrichment for membrane signaling and CUB domain extracellular/plasma membrane-associated proteins (Table 4). This indicates that the mechanisms facilitating refractoriness in stressed mosquitoes rely heavily on differential expression of gene associated with transmembrane activity and the innate immune system. Interesting, while every other module associated with stressed or optimal conditions reflected moderate cross-talk between intramodular hub genes, module E exhibited light associations between hubs (Fig 4A).

**Table 4:** Up to top 4 GO annotation clusters for stress upregulation modules. P-values adjusted by false discovery rate.

Mod	Clstr	GO Biological Process	p-value	GO Molecular Function	p-value	GO Cellular Component	p-value
E	1	GO:0015991~ATP hydrolysis coupled proton transport	0.00049	GO:0046961~proton-transporting ATPase activity, rotational mechanism	0.04192	-	-
E	2	-	-	-	-	GO:0016021~integral component of membrane	0.00419
E	3	GO:0055085~transmembrane transport	0.47292	-	-	-	-
E	4	GO:0006629~lipid metabolic process	0.99921	GO:0052689~carboxylic ester hydrolase activity	0.08903	-	-
F	1	-	-	-	-	GO:0016021~integral component of membrane	0.77858

Modules D, G, and H did not show strong correlations with either the optimal or stressed phenotype and reflected a decrease in gene expression for both treatments from 3 to 18 hours. These are patterns expected of genes representing a common response to DENV infection across both treatments. These modules reflected enrichment in sphingomyelin activity, membrane activity, ATP binding, translation, serine endopeptidase activity and WD40-repeat related protein activity (Table 5).

**Table 5:** Up to top 4 GO annotation clusters for general response modules. P-values adjusted by false discovery rate.

Mod	Clstr	GO Biological Process	p-value	GO Molecular Function	p-value	GO Cellular Component	p-value
D	1	GO:0006685~sphingomyelin catabolic process	0.00029	GO:0004767~sphingomyelin phosphodiesterase activity	0.00018	**-**	**-**
				GO:0016798~hydrolase activity, acting on glycosyl bonds	0.00044		
D	2	**-**	**-**	GO:0005524~ATP binding	0.08599	**-**	**-**
D	3	**-**	**-**	**-**	**-**	GO:0016021~integral component of membrane	0.970066
D	4	**-**	**-**	GO:0004252~serine-type endopeptidase activity	0.99996	**-**	**-**
G	1	GO:0006412~translation	0.03284	GO:0003735~structural constituent of ribosome	0.02833	GO:0005840~ribosome	0.020640
G	2	**-**	**-**	GO:0016887~ATPase activity	0.42381	**-**	**-**
				GO:0005524~ATP binding	0.93935		
G	3	**-**	**-**	GO:0000166~nucleotide binding	0.54683	**-**	**-**
G	4	**-**	**-**	GO:0004252~serine-type endopeptidase activity	0.99999	**-**	**-**
H	1	GO:0006457~protein folding	0.00129	**-**	**-**	GO:0016272~prefoldin complex	0.00114
H	2	**-**	**-**	**-**	**-**	GO:0016021~integral component of membrane	0.99999

Despite lacking a strong correlation to experimental treatment, modules I and J demonstrated striking time-specific differential expression (Table 6). Both modules demonstrate high expression of the optimal treatment and low expression of the stressed treatment at 3 hours, and then a drop in optimal expression and an increase in stressed expression at 18 hours (Fig 2). While neither module I nor J have a strong overall correlation to treatment or time, the reversal of both stressed and optimal expression from 3 to 18 hours shows a time specific pattern for both treatments. Both modules demonstrate enrichment for integral components of membrane function, but module I in particular exhibits extremely strong association with serine-type endopeptidase activity.

**Table 6:** Up to top 4 GO annotation clusters for modules associated with time specific expression. P-values adjusted by false discovery rate.

Mod	Clstr	GO Biological Process	p-value	GO Molecular Function	p-value	GO Cellular Component	p-value
C	1	**-**	**-**	GO:0005524~ATP binding	0.99567	**-**	**-**
C	2	**-**	**-**	**-**	**-**	GO:0016021~integral component of membrane	0.99993
I	1	**-**	**-**	GO:0004252~serine-type endopeptidase activity	0.00001	**-**	**-**
I	2	**-**	**-**	**-**	**-**	GO:0016021~integral component of membrane	0.21746
J	1	-	-	-	-	GO:0016021~integral component of membrane	0.81558

Optimal upregulation modules (A, B, C) and stressed upregulation modules (E, F) exhibit low levels of cross talk, contrasted by high levels of connection between general response modules (D, G, H) and the time specific modules (I, J) (Fig 4B). We anticipate this is due to the effects of environmental conditions during larval rearing subsequently modulating a subset of pre-existing transcriptional factors in the adult female response to DENV exposure, rather than employing completely novel pathways. Full hub gene lists with gene descriptions may be found in Supplemental 3.

### Quantitative PCR validates RNA-seq data

In order to validate our RNA-seq data, we performed qRT-PCR assays. Five genes were chosen from different modules and samples for each permutation of time period (3hr, 18hr), rearing condition (optimal, stressed) and blood meal type (DENV positive, naïve) were examined for differential expression. All five genes exhibited expression patterns consistent with those seen in the normalized RNA-seq data (Supplemental 4).

## Discussion

While much consideration has been given into the differences in susceptibility resulting from genotype by genotype interactions of various populations of *A*. *aegypti* to different DENV serotypes [32, 33], little regard has been given to the impact of the environment in which these interactions normally occur. Here, we hypothesize that the previously reported environmentally induced differences in DENV susceptibility between optimum and stress reared individuals [17] are mediated by differential gene expression. It is important to note that this previous examination of stressed induced refractory phenotype indicated the mechanism of action is not necessarily a midgut escape barrier, as most specimens with midgut infection also demonstrated concurrent disseminated infection [17]; this study employed a whole organism view of the gene expression. Our comparative survey of post DENV-exposure *A*. *aegypti* female gene expression revealed distinct trends associated with stressed and optimal rearing conditions that subsequently determine differences in DENV susceptibility.

During the first 24–48 hours after ingestion of DENV, known as the eclipse period, viral titers have been shown to drop as the midgut epithelia is infected [34, 35], and it is very likely that any barriers to midgut infection would occur during this time period. Dissemination of DENV is typically regarded to occur around three days post exposure, and it has been demonstrated that the midgut epithelial cell may exhibit an infection rate of up to 30% by 2 days post infection [36]. As viral endocytosis and replication in the midgut is known to occur shortly after DENV exposure [18, 19], to examine the associated transcriptional responses we selected early post–infection time points (3h and 18h) which had proven important in previous studies of differential gene expression between refractory and susceptible *A*. *aegpyti* laboratory strains [14].

Our results point to several groups of genes that form distinct modules and work in concert to impact innate immunity. We grouped genes by transcription profiles into modules, allowing us to observe broad, general patterns by co-expression profiles. We envisioned that within these subnetworks there is a subset of major hub genes essential for coordinating a number of pathways responsible for the DENV refractory or susceptible phenotypes. We examined these subnetworks to select critical effectors of phenotype based on degree of network connectivity and selected the top 30 hub genes from the individual modules correlated with the susceptible, optimal phenotype and the refractory, stressed phenotype.

### Optimal upregulation modules

In insects, the innate immune response is a critical effector of pathogen resistance. This resistance occurs through a variety of processes including phagocytosis, secretion of peptides, melanization and physical sequester of pathogens [37]. Several pathways, including but not limited to Toll, JAK-STAT, IMD, and RNAi have been implicated in mosquito response to these pathogens [38, 39]. We previously demonstrated that when stressed during larval development, *A*. *aegypti* exhibit decreased DENV2 dissemination as adults [17], underscoring the importance of environmental background on the innate immune response to foreign pathogens. The current study revealed an association of our susceptible, optimal reared mosquitoes with DNA replication, metabolism, and fatty acid activity. These results largely coincide with the findings of Behura et al. [14], who found a similar over-representation of metabolism and DNA replication in susceptible individuals. It was proposed that these functions may be essential for viral infection [14], noting that the cell cycle environment influences DENV replication in S-phase of C6/36 cells [40]. Further, fatty acid biosynthesis at viral replication sites has been implicated as important for successful DENV replication, with the rate of fatty acid biosynthesis being essential in the cofractionation of lipids with DENV RNA [41, 42]. Together, this suggests that optimal modules (A, B and C) are largely associated with viral co-option of insect pathways to create essential replication complexes by 18 hours post DENV exposure.

A hallmark of these optimally associated modules is a marked increase in expression at 18 hours. As much of the activity is associated with metabolism, it is possible the increased availability of resources in these larger insects may lend to greater viral replication, and may explain why stressed, refractory specimens present lower levels for these transcripts at this time point. If increased metabolism is important for viral replication it is possible that the drop in expression of these genes at 3 hours reflect attempts by the mosquitoes at pre-emptive metabolic regulation to reduce viral replication.

Within these broad associations, a closer examination of optimal modules A, B and C reveal enrichment of autophagy and apoptosis function. For example, module A positively correlates DENV susceptibility with upregulation of caspase 7 (AAEL012143) at 18 hours post infection, confirming the findings of Eng [43] where the silencing of the upstream caspase 7 autophagy initiator *Aedronc* resulted in reduced susceptibility to DENV infection. The apoptosis/autophagy contributor cathepsin B (AAEL007590) [44] and an autophagy related gene ATG4B (AAEL007228), both from module B, and an autophagy related hub gene APG8 (AAEL007162), from module C, lend further credence to the importance of autophagy to the susceptible phenotype and aligns with past studies that suggest the regulation of lipid metabolism in DENV induced autophagy [45].

### Stress upregulation modules

The importance of cell membrane recognition, endocytosis and endosome regulation towards pathogen resistance is well documented [18, 19]. Enrichment of our stress associated modules revealed heavy involvement of protein transmembrane transport and signaling pathways. The Janus kinase (JAK/STAT) and Toll pathways have demonstrated importance in regulation of innate immunity to viruses in *Drosophila* [46, 47]. While the mechanism of action is largely unknown, both pathways utilize RNAi mechanisms in order to confer limited viral resistance in *Drosophila* [38, 48]. Both the JAK/STAT and Toll pathways have demonstrated high levels of conservation of function and form between flies and mosquitoes [49] and are known to be of particular significance to *A*. *aegypti* DENV resistance [12]. Analysis of our data associates stress module E with the upregulation of JAK/STAT and Toll genes that have been previously implicated in the innate immune response of *A*. *aegypti* to DENV. The first gene of interest, Toll9A (AAEL013441), was shown to be upregulated in mosquito carcasses and midguts in response to DENV exposure, as well after silencing of the negative Toll regulators Cactus and Caspar [12]. The second, DENV restriction factor (DVRF1) (AAEL008492), is a membrane bound downstream target of the JAK/STAT pathway that has been implicated in genetic predilection towards DENV resistance [13, 50]. Additionally, upregulation of a WD40-repeat protein, Ukn7703 (AAEL007703), in module F was also associated with stress mediated DENV resistance [51]. WD40-repeat proteins are known to be involved in a range of functions including signal transduction, transcriptional control, cell cycle control, autophagy and apoptosis. Although WD40-repeat proteins present this wide range of functions, a recent study demonstrated that silencing of the Ukn7703 signaling protein resulted in overexpression of the Janus kinase Hopscotch and JAK/STAT receptor Domeless, and subsequent DENV inhibition [50]. The same study also examined the aforementioned DVRF1, and found that it was not impacted by the upregulation of the upstream Hopscotch and Domeless genes and concluded there must be a limiting factor downstream of the latter two genes. The differential expression of Toll9A, DVRF1 and Ukn7702 confirm the importance of the Toll and JAK/STAT pathways to innate immune responses triggered by DENV exposure and further indicate that the level of response is further modulated by environmental conditioning. That said, the conspicuous absence of differential expression in our data of key pathway contributors such as Domeless, Hopscotch, Spaetzle, Cactus, Re1A, and Tep13 suggest that environmental stress may utilize modified pathways. This is supported by the high number of network connections revealed by a STRING database search on predicted functional gene partners—Toll9A presented 10 experimentally determined edges, and DVRF presented 8 experimental edges.

The association of DENV refractoriness with protein transmembrane transport and neuropeptide signaling is in line with previous assertions that the mechanism of action for environmentally stressed, refractory mosquitoes is more likely an early midgut infection barrier rather than a physical midgut escape barrier [17]. Enrichment of the myosin complex supports the likelihood of it being a key player in transporting endocytic vesicles from the plasma membrane to the cytosol [52–54]. Specifically, calponin/transgelin (AAEL008315) and its downstream target calmodulin (AAEL006921) [55] are hub genes in modules E and F, respectively. Further, as previous studies have demonstrated calponin associated dephosphorylation inhibits myosin-associated activity [56, 57], it is likely that its upregulation in refractory mosquitoes interferes with the proper egress of DENV into the cytosol. Calmodulin exhibits high levels of intramodular connectivity and verifies the importance of major hub genes such as calponin and cross-talk between modules. At this point it is important to note that the modules correlated with the refractory, stressed phenotype (E, F) both reflect high upregulation in stressed samples at 3 hours and down regulation of optimal expression at both time points. This can be contrasted with the modules associated with DENV susceptibility (A, B, C) in which genes reflect conserved high upregulation in optimal 18 hour samples. Together, this underscores the importance of time specific expression to the refractory and susceptible phenotypes.

Unexpectedly, the refractory, stressed module E also contains seven vacuolar ATPase (vATPase) subunits (AAEL002917, AAEL008787, AAEL005798, AAEL006516, AAEL011025, AAEL012035, AAEL012113) previously associated with DENV susceptibility in a study comparing immune response of mosquitoes of divergent genetic background [14]. Additionally, four of the vATPases associated with module E (AAEL012819, AAEL011025, AAEL012113, AAEL002464) resulted in reduced DENV infection when knocked down in other studies [58, 59]. In contrast, a vATPase (AAEL015594) from the first study [14] was associated with our susceptible, optimal module B, and another vATPase (AAEL006390), not found in other DENV studies, was found in module A. It is widely accepted that vATPases are critical for the acidification of endosomes necessary for the egress of dengue virus [14, 19]. While this seems to be at odds with our current study, it is important to note that previous studies examined differences in susceptibly due to divergent genetic background, while here we examined gene-environment interactions in sibling mosquitoes reared under different conditions. Again, STRING analyses reveal that each of these vATPases have numerous documented interactions and may have disparate modes of action resulting from varied animal and viral genetic backgrounds in the greater context of adaptive plasticity.

Previously, Price et al. concluded that nutritionally induced stress should increase innate immunity to viral infection in DENV exposed mosquitoes [60], a hypothesis that was confirmed by the observed association with classic JAK/STAT and Toll pathway genes in our current study. The same study by Price et al., reported reduced vitellogenesis and increased expression of transcripts related to transmembrane activity in naïve blood fed stressed mosquitoes [60]. Similarly, our module E exhibited increased transmembrane activity and included an allatostatin (AAEL012139), a neuropeptide known to inhibit juvenile hormone mediated vitellogenesis in insects [61–65], as a major hub gene of this stress associated module. At this point, it is important to note that a key difference between the aforementioned studies is our use of naïve or DENV infected blood meals. Our results were screened for significantly higher expression of our DENV exposed treatments when compared to our naïve blood fed controls. This indicates that while nutritional stress may prime mosquito expression for these traits, DENV exposure triggers significant upregulation of these transcripts.

### Conserved response modules and modules demonstrating inverted expression over time

Modules D, G and H, demonstrated sharp drops in expression over time for both stressed and optimal treatments. Interestingly, modules D and G both exhibit high enrichment for WD40 motifs similar to those seen in Ukn7703 in module F. This is unsurprising and confirms our suspicions that many responses are conserved in both stressed and optimal DENV responses.

In contrast, module I presented enrichment for both serine protease and endopeptidase inhibitor activity, with the opposing differential expression patterns of both the optimal and stressed samples transposing from 3 hours to 18 hours. While the exact role of serine protease activity remains unclear [15, 16], it is apparent that these proteins are involved in functions that exhibit time and treatment specific differential expression in response to dengue exposure. These results are supported by a previous study demonstrating a positive correlation between an early fatty acid synthase (AAEL001194) and proliferation of DENV in the *Aedes aegypti* midgut 4 days post infection [42]. Specifically, the initial high expression of this fatty acid in the optimal treatment provides further evidence that differences between optimal and stressed dissemination rates are due to mechanisms related to early viral replication rather than a midgut escape barrier.

Interestingly, modules I and J, in which stressed and optimal expression swapped over time, demonstrated the most global-cross talk—especially with each other, the conserved response modules (D, G, H) and optimal module C. This suggests that the activity demonstrated in module I may indeed result in an overall increase in susceptibility to dengue. Whether these expression patterns are the result or the cause of the mechanism responsible for stress-conditioned differences of susceptibility remains to be seen.

### Conclusion

Different populations of *A*. *aegypti* are known to exhibit variable levels of competence to host different DENV isolates based on genotypes of both the mosquito and the virus. The literature is further confounded by studies offering seemingly conflicting results on the impact of stress on mosquito susceptibility to different arboviruses. Our previous findings on stress reducing susceptibility of *A*. *aegypti* to DENV2 [17] correspond to the results found in wild *A*. *aegypti* [66], but generally contradict a study reporting significantly lower DENV2 infection rates in larger *A*. *albopictus* and no size related effects in a laboratory strain of *A*. *aegypti* [9]. This is further complicated by the inclusion of literature for other arbovirus species, suggesting that larval stress increases the susceptibility *A*. *aegypti* to Sindbis virus (SINV) [67, 68]. It is possible that much of these differences may be explained by species specific responses to different strains of arboviruses, or that the laboratory strains may have lost phenotypic plasticity over 80 years of captivity [69]. As such, our experimental design eliminates differences in genetic architecture and arbovirus strains and focuses on the impact of environmental stress on the susceptibility of a recent field isolate of *A*. *aegypti* susceptibility to a single isolate of DENV2. Our data indicates that there exists a wide array of transcriptional responses unique to DENV exposure when compared to naïve blood meals. These responses were not limited to classic innate immunity pathways, and underscore the incomplete nature of our current understanding of immunity in mosquitoes. Here, based on a mechanism stemming from environmental stress, we present full gene lists of ten modules, representing co-expression networks correlated with DENV susceptibility (A, B, C), DENV refractoriness (E, F), general DENV response (D, G, H) and time specific DENV response (I, J), as well as the top 30 master regulatory or hub genes that show high connectivity in each module. While qRT-PCR validation supports the reliability of these correlations, the chief purpose of this study is to serve as a platform for further mechanistic and functional analyses. In the future, we plan to perform RNA interference knock-downs of these candidate genes in these mosquitoes, as well as laboratory strains with documented differences in DENV susceptibility, to see how strongly these individual genes influence the phenotype. Additionally, we expect to expand these future studies to include tissue and time-specific analyses. We anticipate that further functional analyses of these genes may lead to novel forms of DENV control and inform on which mechanisms of the immune response may be conserved in the field independent of environmental conditions.

## Supporting information

S1 Table Full gene lists delimited by module. (XLSX)

S2 Table Full gene ontology (GO) annotations delimited by module. (XLSX)

S3 Table Full hub gene lists delimited by module. (XLSX)

S1 File qRT-PCR validation. Expression via relative quantitation to RPS17 endogenous control.
(XLSX)
